# High Fibrinogen to Albumin Ratio: A Novel Marker for Risk of Stroke-Associated Pneumonia?

**DOI:** 10.3389/fneur.2021.747118

**Published:** 2022-01-13

**Authors:** Gangqiang Lin, Minlei Hu, Jiaying Song, Xueqian Xu, Haiwei Liu, Linan Qiu, Hanyu Zhu, Minjie Xu, Dandan Geng, Lexuan Yang, Guiqian Huang, Jincai He, Zhen Wang

**Affiliations:** ^1^Department of Neurology, The First Affiliated Hospital of Wenzhou Medical University, Wenzhou, China; ^2^Department of Neurology, The First Hospital of Jiaxing, Jiaxing, China; ^3^School of Mental Health, Wenzhou Medical University, Wenzhou, China

**Keywords:** acute ischemic stroke, stroke-associated pneumonia, coagulation, inflammation, fibrinogen, albumin

## Abstract

**Background:** Stroke-associated pneumonia (SAP) is associated with poor prognosis after acute ischemic stroke (AIS).

**Purpose:** This study aimed to describe the parameters of coagulation function and evaluate the association between the fibrinogen-to-albumin ratio (FAR) and SAP in patients with AIS.

**Patients and methods:** A total of 932 consecutive patients with AIS were included. Coagulation parameters were measured at admission. All patients were classified into two groups according to the optimal cutoff FAR point at which the sum of the specificity and sensitivity was highest. Propensity score matching (PSM) was performed to balance potential confounding factors. Univariate and multivariate logistic regression analyses were applied to identify predictors of SAP.

**Results:** A total of 100 (10.7%) patients were diagnosed with SAP. The data showed that fibrinogen, FAR, and D-dimer, prothrombin time (PT), activated partial thromboplastin time (aPTT) were higher in patients with SAP, while albumin was much lower. Patients with SAP showed a significantly increased FAR when compared with non-SAP (*P* < 0.001). Patients were assigned to groups of high FAR (≥0.0977) and low FAR (<0.0977) based on the optimal cut-off value. Propensity score matching analysis further confirmed the association between FAR and SAP. After adjusting for confounding and risk factors, multivariate regression analysis showed that the high FAR (≥0.0977) was an independent variable predicting the occurrence of SAP (odds ratio =2.830, 95% CI = 1.654–4.840, *P* < 0.001). In addition, the FAR was higher in the severe pneumonia group when it was assessed by pneumonia severity index (*P* = 0.008).

**Conclusions:** High FAR is an independent potential risk factor of SAP, which can help clinicians identify high-risk patients with SAP after AIS.

## Introduction

Stroke-associated pneumonia (SAP) is one of the most common complications among patients with acute ischemic stroke (AIS), taking place most frequently within the first week of stroke onset (1–3). The incidence of SAP ranges from 6.7 to 37.98% (4–6). Patients with SAP are more likely to have a worse outcome than patients with non-SAP, namely, poor functional prognosis, excessive time in hospital, and an increased risk of disability, 30-day and 1-year mortality (7–9). In addition, a retrospective cohort study including 14,702 patients with AIS found that pneumonia was significantly associated with the development of non-pneumonia medical complications, such as gastrointestinal bleeding, urinary tract infection, and recurrent stroke (10). Several clinical trials have shown that prophylactic use of antibiotics was ineffective to prevent SAP (11, 12).

Numerous studies have discovered various risk factors for SAP such as old age, being male, stroke severity, dysphagia, and diabetes (5, 9, 13). Moreover, researchers created several predictive models with these risk factors (4, 14–17), among which the A2DS2 score [age, atrial fibrillation (AF), dysphagia, sex, and severity] was considered to have a good predictive capacity (18). In a consecutive cohort of 1,569 patients with AIS, Gong et al. proved that the A2DS2 score could effectively predict the development of SAP in the Chinese population (19). However, SAP prediction is still a challenge due to its atypical clinical symptoms and the low accuracy of X-ray images (20). Therefore, it is necessary for us to look for valid predictors to diagnose SAP as early as possible.

It has long been known that there exists a wide range of interplay between inflammation and coagulation. For instance, inflammation spikes immediately after injury. Aggregated platelets and neutrophils release factors that stimulate the coagulation cascade in acute lung disease (21).

The fibrinogen-to-albumin ratio (FAR) which combines coagulation with nutritional status, is a new vital inflammatory biomarker for a variety of diseases, such as cervical cancer, oligodendroglial gliomas, acute coronary syndrome, and stroke (22–25). Zheng et al. found that a high FAR level on admission was highly associated with 3-month mortality and disability in patients with acute lacunar stroke (22). Yet, the link between FAR and the risk of developing SAP in patients after AIS remains unclear. The pneumonia severity index (PSI) is one of the best-known predictors for prognosis, containing 20 variables covering demography, clinical features, physical examination, laboratory examination, and chest radiography (26). A decade after PSI was founded, Aujesky et al. proved that PSI could effectively predict mortality and other adverse outcomes of low-risk patients and provided useful feedback to guide initial treatment (27).

Here, we aimed to explore the relationship between SAP and FAR in a retrospective cohort, which might provide an easily available and economical predictor for SAP.

## Materials and Methods

### Study Population

Patients were collected from a retrospective clinical database that included consecutive patients admitted to the Department of Neurology, First Affiliated Hospital of Wenzhou Medical University, within 24 h after the onset of ischemic stroke between March 2018 and January 2019 in this retrospective study. This retrospective study obtained the approval of the Ethics Committee of the First Affiliated Hospital of Wenzhou Medical University and was conducted according to the ethical standards of the local Research Ethics Committee on human experimentation.

**Figure 1:**
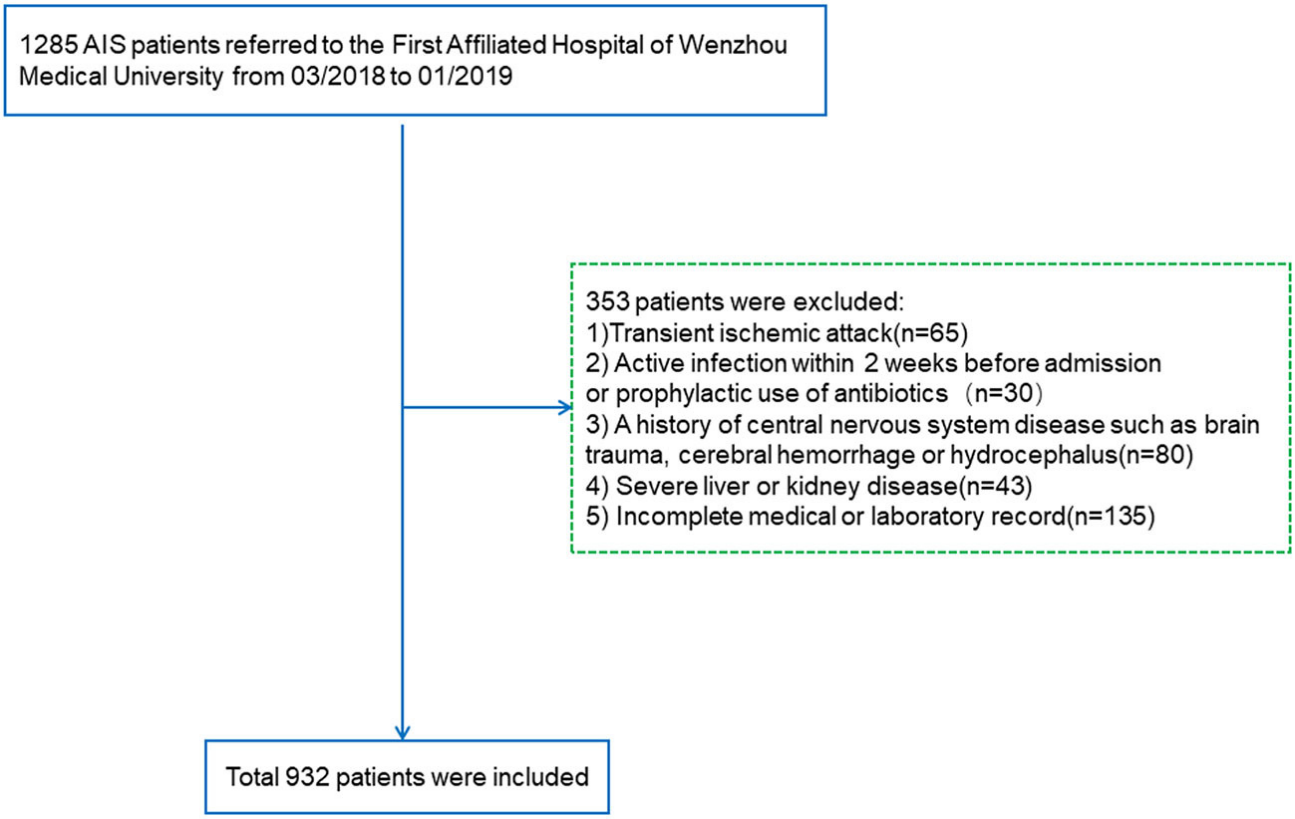
Research flowchart. AIS, acute ischemic stroke.

Included patients diagnosed with AIS were verified with CT or MRI within 24 h at admission. The exclusion criteria were as follows (i) transient ischemic attack; (ii) active infection within 2 weeks before admission or prophylactic use of antibiotics; (iii) a history of central nervous system diseases such as brain trauma, cerebral hemorrhage, or hydrocephalus; (iv) severe liver disease [serum transaminase levels more than twice the upper limit of the normal range or persistent hyperbilirubinemia within 6 months]; (v) severe kidney disease [estimated glomerular filtration rate (eGFR) < 60 ml/min/1.73 m^2^]; and (vi) incomplete medical or laboratory record. Ultimately, a total of 932 patients were enrolled in this study (Figure 1).

### Data Collection

Demographic data included age, gender, history of previous disease, smoking, and drinking. Pre-existing comorbidities included hypertension, diabetes mellitus, coronary artery disease, AF. Other clinical variables collected prescriptions during hospital (antiplatelet, anticoagulation, stain, and thrombolysis), laboratory tests [fibrinogen, platelets, prothrombin time (PT), activated partial thromboplastin time (aPTT), international normalized ratio (INR), D-dimer, and albumin], and blood pressure measurements were conducted within 24 h of hospital admission. Stroke severity, evaluated using the National Institutes of Health Stroke Scale at admission were assessed by well-trained neurologists. Functional outcomes were assessed by the modified Rankin Scale (mRS) at discharge. The same occupational neurologist evaluated swallowing function in all participants at baseline with the modified water swallowing test. The assessment mainly consisted of behavioral airway response, especially coughing, choking, or throat clearing, and/or change in voice (28). The PSI, a well-verified scoring system of pneumonia burden, was used to assed the severity of pneumonia in the SAP participants (29).

### Assessment of Outcome

Stroke-associated pneumonia was defined as lower respiratory tract infections within the first 7 days after stroke onset, accordance to the modified Center for Disease Control and Prevention criteria (30). Patients were diagnosed with SAP based on clinical symptoms, laboratory examination, and confirmation by chest X-ray and CT (14). Furthermore, the diagnosis of SAP was conducted by two well-trained neurologists blinded to the data of the patients. This study only recorded in-hospital pneumonia; community-acquired pneumonia and pneumonia before the stroke were excluded from consideration.

### Propensity-Score Matching

We used propensity-score matching to balance confounding factors in baseline characteristics between the low FAR group and high FAR group. The propensity score was estimated using logistic regression of independent variables for each patient that included age, current smoking, drinking, NHISS, previous stroke, diabetes mellitus, AF, thrombolysis, dysphagia, aPTT, D-dimer, PLT, length of hospital stay, and discharge mRS score. If the propensity scores of patients were the same, they had an equal probability of encountering events that we wanted to observe. We adopted 1:1 propensity score-matching to match the low FAR group and high FAR group.

### Statistical Analysis

Continuous variables were presented as mean with standard deviation or median with interquartile range according to normal or asymmetrical distribution, while discrete variables were shown as frequencies or percentages. Data normality was checked with Kolmogorov–Smirnov test. Continuous variables in normal distribution were compared by Student's *t*-test, while asymmetrically distributed variables were analyzed by Mann–Whitney U-test, and comparison of proportions were analyzed by the chi-squared test. The relationship between FAR and SAP was analyzed in four steps. First, we conducted a receiver operating characteristic (ROC) curve to determine the optimal value of the area under the curve (AUC), and a significant cutoff point, sensitivity, and specificity. We considered the optimal cutoff point by calculating Youden's index max point giving the highest sum of sensitivity and specificity and maximum diagnostic efficiency. Second, binary logistic regression was performed to explore the relationship between this FAR cutoff point and SAP. Third, all confounding factors (*P* < 0.05) were included in multiple logistic regression to adjust for potential confounding factors and find out the independent factors for SAP. Finally, we compared FAR levels between the severe SAP group and non-severe SAP group divided by PSI. All statistical analyses were performed with SPSS for Windows, version 25.0 (SPSS Inc., Chicago, IL, USA). All the statistics are two-tailed and *P* < 0.05 was considered statistically significant.

## Results

### Baseline of Characteristics of Patients in SAP Group and Non-SAP Group

During the research period, 932 patients with AIS were included in the study. The baseline demographic, clinical, and laboratory characteristics of the study population are displayed in Table 1. A total of 593 (63.0%) patients were male, and the mean age of the enrolled patients was 67.0 years (59.0–74.0 years). Compared with patients with non-SAP, SAP patients who were older (*P* < 0.001), had higher National Institute of Health Stroke Scale (NIHSS) scores at admission (*P* < 0.001), higher mRS scores at discharged and longer hospital stay, were more likely to acquire dysphagia (*P* = 0.031). Besides, patients with SAP were more likely to receive anticoagulation treatment and were less likely to undergo antiplatelet therapies.

**Table 1:** Baseline of characteristics of patients in SAP group and non-SAP group.

**Variables**	**All patients**	**Non-SAP (*n* = 832)**	**SAP (*n* = 100)**	***P*** **value**
FAR	0.089 (0.075–0.109)	0.087 (0.074–0.105)	0.113 (0.090–0.167)	<0.001
Age (years)	67.0 (59.0–74.0)	66.0 (59.0–73.0)	72.0 (62.5–80.0)	<0.001
Male	596 (63%)	533 (64.1%)	63 (63.0%)	0.834
Current smoking	376 (40.3%)	338 (40.6%)	38 (38.0%)	0.613
Drinking	350 (37.6%)	314 (37.7%)	36 (36.0%)	0.734
Baseline SBP	151.5 (136.0–168.0)	151.0 (136.0–168.0)	152.0 (138.0–168.0)	0.475
Baseline DBP	82.0 (74.0–92.0)	82.0 (74.0–92.0)	83.0 (77.0–94.5)	0.267
NHISS	3.0 (1.0–6.0)	2.0 (1.0–5.0)	9.5 (3.5–13.0)	<0.001
Previous stroke	133 (14.3%)	112 (13.5%)	21 (21.0%)	0.042
Hypertension	713 (76.5%)	635 (76.3%)	78 (78.0%)	0.708
Diabetes mellitus	369 (39.6%)	338 (40.6%)	31 (31.0%)	0.063
CAD	20 (2.2%)	19 (2.3%)	1 (1.0%)	0.401
AF	111 (11.9%)	83 (10.0%)	28 (28.0%)	<0.001
Dysphagia	129 (13.8%)	74 (8.9%)	55 (55.0%)	<0.001
Antiplatelet drugs	883 (93.7%)	798 (95.9%)	85 (85.0%)	<0.001
Anticoagulant drugs	129 (13.8%)	108 (13.0%)	21 (21.0%)	0.028
Stains	920 (98.7%)	823 (98.9%)	97 (97.0%)	0.108
Thrombolysis	37 (4.0%)	32 (3.9%)	5 (5.0%)	0.580
Stroke etiology				<0.001
Atherosclerosis	725 (77.9%)	658 (79.2%)	67 (67.0%)	
Cardioembolism	94 (1.0%)	3 (0.4%)	0 (0.0%)	
Small vessel occlusion	122 (13.1%)	94 (11.3%)	28 (28.0%)	
Other causes	18 (2.0%)	17 (2.0%)	1 (1.0%)	
Length of hospital stay	9.97 (8.0–12.0)	9.0 (8.0–11.0)	11.0 (9.0–14.0)	<0.001
Discharge mRS score	2.0 (1.0–3.0)	2.0 (1.0–2.0)	4.0 (2.0–4.0)	<0.001

*SAP, stroke-associated pneumonia; SBP, systolic blood pressure; DBP, diastolic blood pressure; NIHSS, National Institute of Health Stroke Scale; CAD, coronary artery disease; AF, atrial fibrillation; mRS, modified Rankin Scale*.

### Comparison of Initial Indexes of Coagulation Function Between Patients With SAP and Non-SAP

**Figure 2:**
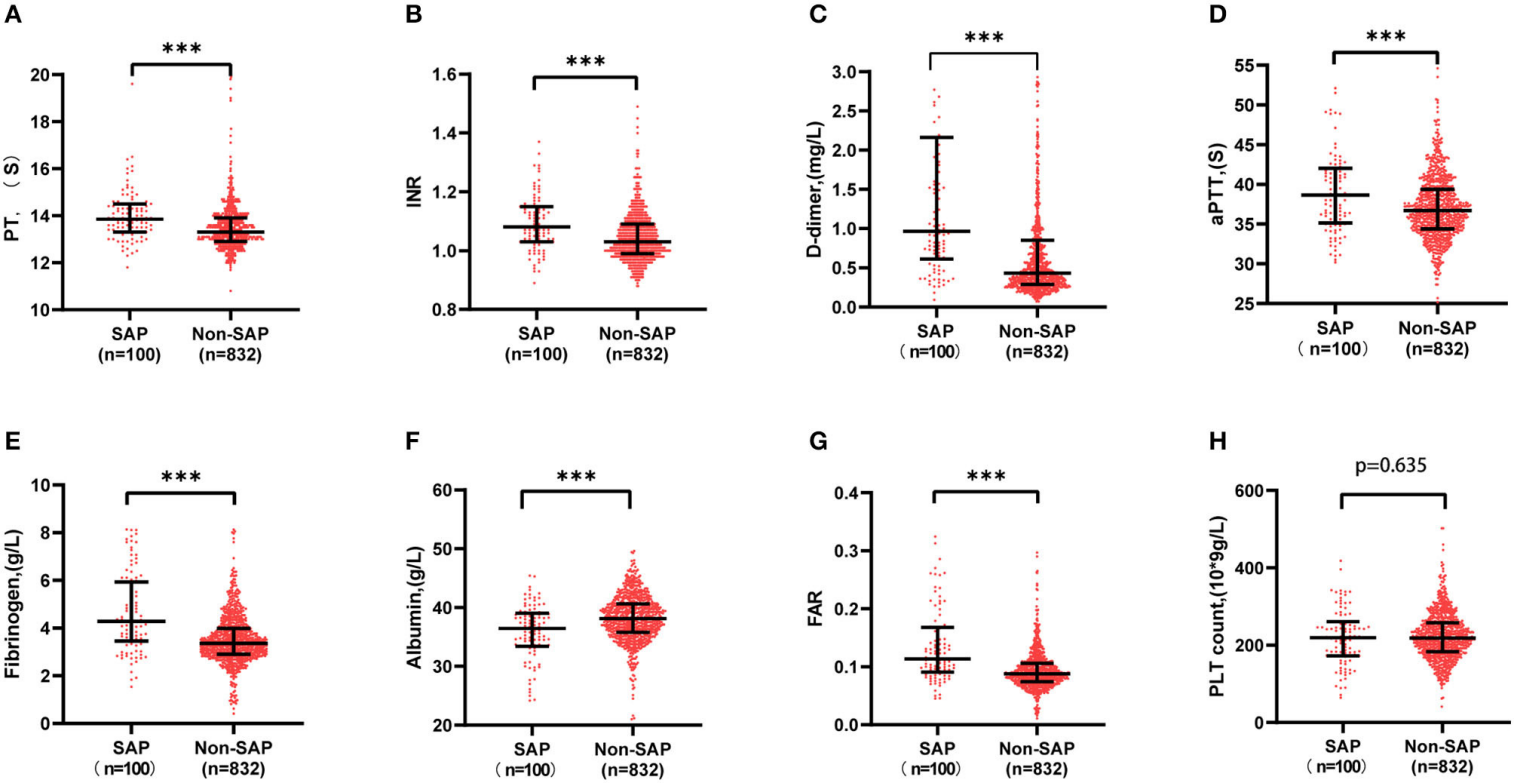
Comparison of initial indexes of coagulation function between SAP and non-SAP patient. Fibrinogen, FAR, PT, INR, aPTT, and D-dimer were significantly higher in the SAP group than in the non-SAP group (4.23 vs. 3.07 g/l, 0.113 vs. 0.087, 13.85 vs. 13.3 s, 1.08 vs. 1.03, 38.65 vs. 36.70 s, 0.96 vs. 0.43 mg/l), while albumin was much lower (36.45 vs. 38.1 g/l). ***P < 0.001. aPTT, activated partial thromboplastin time; FAR, fibrinogen to albumin ratio; INR, international normalized ratio; PT, prothrombin time; SAP, stroke-associated pneumonia.

It was shown that fibrinogen, FAR, PT, INR, aPTT, and D-dimer were significantly higher in the SAP group than in the non-SAP group (4.23 vs. 3.07 g/l, 0.113 vs. 0.087, 13.85 vs.13.3 s, 1.08 vs. 1.03, 38.65 vs. 36.70 s, 0.96 vs. 0.43 mg/l, *P* < 0.001, respectively), while albumin was much lower (36.45 vs. 38.1 g/l, *P* < 0.001), as shown in Figure 2.

### Baseline Characteristics of All Patients in the High and Low FAR Groups

**Figure 3:**
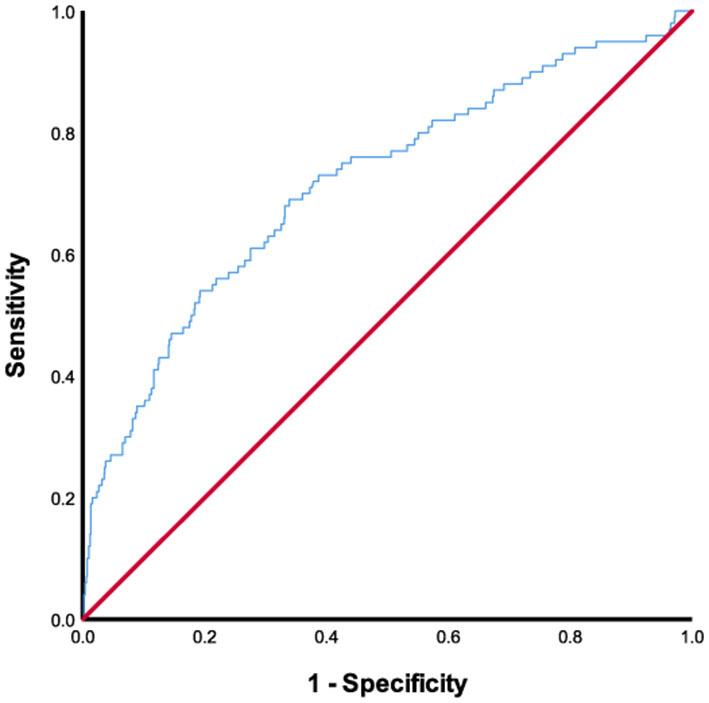
ROC analysis of fibrinogen-to-albumin ratio for predicting stroke-associated pneumonia. ROC, receiver operating characteristic.

The ROC curve for FAR showed an AUC of 0.717 (95% CI, 0.659–0.775; *P* < 0.001) and pointed to an optimal cutoff level of 0.0977 mg/dl of FAR with 69.0% sensitivity and 66.1% specificity for the development of SAP during hospitalization (Figure 3). The patients were assigned to two groups [high FAR (≥0.0977) or low FAR (<0.0977)]. The characteristics of the AIS participants between the two FAR groups, namely, the demographic, clinical, and laboratory characteristics, are depicted in Table 2. As shown in Table 2, patients with higher FAR levels were older; were more likely to be diabetic; had higher NIHSS scores at admission, length of stay, and mRS scores at discharge; and had higher fibrinogen, aPTT, D-dimer, and PLT counts, and lower albumin levels.

**Table 2:** Baseline characteristics of all patients in the high (≥0.0977) and low FAR (<0.0977) groups.

**Variables**	**All patients**	**Low FAR (*n* = 580)**	**High FAR (*n* = 352)**	***P*** **-value**
SAP	100 (10.7%)	31 (5.3%)	69 (19.7%)	<0.001
Age (years)	67.0 (59.0–74.0)	65.0 (57.0–72.0)	69.0 (62.0–77.0)	<0.001
Male	596 (63%)	376 (64.7%)	220 (62.7%)	0.530
Current smoking	376 (40.3%)	227 (39.1%)	149 (42.5%)	0.308
Drinking	350 (37.6%)	232 (39.9%)	118 (33.6%)	0.054
Baseline SBP	1,515 (136.0–168.0)	151 (137.0–168.0)	151 (136.0–168.0)	0.939
Baseline DBP	82.0 (74.0–92.0)	83.0 (75.0–92.0)	82.0 (74.0–91.0)	0.377
NHISS	3.0 (1.0–6.0)	2.0 (1.0–5.0)	4.0 (1.0–8.0)	<0.001
Previous stroke	133 (14.3%)	67 (11.5%)	65 (18.8%)	0.002
Hypertension	713 (76.5%)	442 (76.1%)	271 (77.2%)	0.693
Diabetes mellitus	369 (39.6%)	208 (35.8%)	161 (45.9%)	0.002
CAD	20 (2.2%)	14 (2.4%)	6 (1.7%)	0.470
AF	111 (11.9%)	60 (10.3%)	52 (14.8%)	0.041
Dysphagia	129 (13.8%)	50 (8.6%)	79 (22.5%)	<0.001
Antiplatelet drugs	883 (93.7%)	555 (95.5%)	328 (93.4%)	0.169
Anticoagulant drugs	129 (13.8%)	72 (12.4%)	57 (16.4%)	0.099
Stains	920 (98.7%)	575 (99.0%)	345 (98.3%)	0.375
Thrombolysis	37 (4.0%)	30 (5.2%)	7 (2.0%)	0.016
Fibrinogen	3.62 (2.93–4.12)	3.06 (2.74–3.36)	4.43 (3.89–5.11)	<0.001
PT	13.59 (12.90–14)	13.40 (12.90–14.00)	13.40 (13.00–14.00)	0.310
INR	1.05 (0.99–1.10)	1.03 (0.99–1.09)	1.04 (0.99–1.10)	0.283
aPTT	37.37 (34.50–39.80)	36.2 (34.30–38.8)	37.5 (34.8–40.92)	<0.001
D-dimer	0.79 (0.30–0.96)	0.38 (0.28–0.76)	0.67 (0.38–1.15)	<0.001
Albumin	37.83 (35.50–40.50)	39.10 (37.00–41.60)	35.70 (33.80–38.42)	<0.001
PLT	218.0 (181.0–258.0)	214.0 (180.0–248.0)	226.0 (186.0–273.0)	0.001
Stroke etiology				0.247
Atherosclerosis	725 (77.9%)	456 (78.5%)	269 (76.9%)	
Cardioembolism	94 (1.0%)	67 (11.5%)	55 (15.7%)	
Small vessel occlusion	122 (13.1%)	41 (7.1%)	21 (6.0%)	
Other causes	18 (2.0%)	14 (2.4%)	4 (1.2%)	
Length of hospital stay	9.97 (8.0–12.0)	9.0 (7.0–11.0)	9.0 (8.0–13.0)	<0.001
Discharge mRS score	2.0 (1.0–3.0)	2.0 (1.0–2.0)	2.0 (1.0–3.0)	<0.001

*SAP, stroke-associated pneumonia; SBP, systolic blood pressure; DBP, diastolic blood pressure; INR, international normalized ratio; NIHSS, National Institute of Health Stroke Scale; CAD, coronary artery disease; AF, atrial fibrillation; PT, prothrombin time; aPTT, activated partial thromboplastin time; mRS, modified Rankin Scale*.

As shown in Table 3, after propensity score matching (PSM), 277 patients with high FAR levels were individually 1:1 matched to 277 patients with low FAR levels. The results above remained significant after controlling for the confounders, namely, fibrinogen, D-dimer, PLT counts, albumin, and the number of patients with SAP.

**Table 3:** Baseline characteristics of all patients in the high (≥0.0977) and low FAR (<0.0977) groups after propensity score matching.

**Variables**	**All patients**	**Low FAR (*n* = 277)**	**High FAR (*n* = 277)**	***P*** **-value**
SAP	67 (12.1%)	23 (8.3%)	44 (15.9%)	0.006
Age(years)	68.0 (61.0–76.0)	69.0 (62.0–75.0)	68.0 (61.0–76.0)	0.644
Male	211 (38.1%)	104 (37.5%)	107 (38.6%)	0.793
Current smoking	218 (39.4%)	104 (37.5%)	114 (41.2%)	0.384
Drinking	191 (34.5%)	94 (33.9%)	97 (35.0%)	0.789
Baseline SBP	152 (137.0–168.0)	155.0 (139.0–169.0)	151.0 (135.0–167.0)	0.100
Baseline DBP	82.0 (74.0–91.0)	82.0 (75.0–92.0)	82.0 (73.0–91.0)	0.677
NHISS	3.0 (1.0–7.0)	3.0 (1.0–7.0)	3.0 (1.0–7.0)	0.331
Previous stroke	82 (14.8%)	41 (14.8%)	41 (14.8%)	1.000
Hypertension	416 (75.1%)	208 (75.1%)	208 (75.1%)	1.000
Diabetes mellitus	226 (40.8%)	102 (36.8%)	124 (44.8%)	0.057
CAD	13 (2.4%)	8 (2.9%)	5 (1.8%)	0.392
AF	83 (15.0%)	41 (14.8%)	42 (15.2%)	0.905
Dysphagia	521 (94.0%)	260 (93.9%)	261 (94.2%)	0.858
Antiplatelet drugs	82 (14.8%)	40 (14.4%)	42 (15.2%)	0.811
Anticoagulant drugs	546 (98.6%)	275 (99.3%)	271 (97.8%)	0.154
Stains	18 (3.2%)	11 (4.0%)	7 (2.5%)	0.338
Thrombolysis	84 (15.2%)	43 (15.5%)	41 (14.8%)	0.813
Fibrinogen	3.63 (3.08–4.35)	3.1 (2.8–3.41)	4.35 (3.86–5.01)	<0.001
PT	13.4 (13.0–14.0)	13.5 (12.9–14.0)	13.4 (13.0–13.9)	0.705
INR	1.04 (1.00–1.10)	1.04 (1.00–1.10)	1.04 (0.99–1.09)	0.696
APTT	36.95 (34.6–40.0)	36.5 (34.7–39.8)	37.2 (34.5–40.4)	0.456
D-dimer	0.51 (0.31–0.97)	0.43 (0.28–0.76)	0.61 (0.36–1.06)	<0.001
Albumin	37.50 (35.10–40.10)	38.90 (36.80–41.30)	36.00 (34.00–38.95)	<0.001
PLT	227.0 (192.0–265.2)	233.0 (198.0–266.5)	218 (181.0–263.0)	0.026
Stroke etiology				0.818
Atherosclerosis	421 (76.1%)	213 (76.9%)	208 (75.4%)	
Cardioembolism	88 (15.9%)	43 (15.5%)	45 (16.3%)	
Small vessel occlusion	34 (6.1%)	16 (5.8%)	18 (6.5%)	
Other causes	10 (3.8%)	5 (1.9%)	5 (1.9%)	
Length of hospital stay	9 (8.0–12.0)	9.0 (8.0–12.0)	9.0 (8.0–12.0)	0.555
Discharge mRS score	2.0 (1.0–3.0)	2.0 (1.0–3.0)	2.0 (1.0–3.0)	0.492

*SAP, stroke-associated pneumonia; SBP, systolic blood pressure; DBP, diastolic blood pressure; INR, international normalized ratio; NIHSS, National Institute of Health Stroke Scale; CAD, coronary artery disease; AF, atrial fibrillation; PT, prothrombin time; aPTT, activated partial thromboplastin time; mRS, modified Rankin Scale*.

### Association Between the Level of FAR and SAP

Table 4 showed the regression models for the association between FAR and SAP. After adjusting for potential confounders, FAR ≥ 0.0977 was an independent predictive factor for higher risk of SAP [odds ratio (OR) 2.830; 95% CI 1.654–4.840]. Moreover, age, aPTT, dysphagia, and NIHSS on admission were significantly associated with SAP in AIS patients (binary logistic regression, OR 1.031, 95% CI 1.007–1.056; *P* = 0.011; OR 1.074, 95% CI 1.021–1.130; *P* = 0.005; OR 5.216, 95% CI 2.953–9.212; *P* < 0.001; OR 1.138 95% CI 1.076–1.203, respectively) (Table 4).

**Table 4:** Multivariate logistic models for risk factors of SAP.

	**Unadjusted analysis**		**Adjusted analysis**	
	**OR (95% CI)**	***P*** **-value**	**OR (95%CI)**	***P*** **-value**
Age	1.059 (1.037–1.081)	<0.001	1.031 (1.007–1.056)	0.011
Sex, female	1.047 (0.681–1.609)	0.834		
NIHSS on admission	1.251	<0.001	1.138 (1.076–1.203)	<0.001
Atrial fibrillation	3.308 (2.038–5.370)	<0.001	NS	NS
Dysphagia	12.520 (7.898–19.845)	<0.001	5.216 (2.953–9.212)	<0.001
Albumin^a^	0.886 (0.848–0.927)	<0.001		
PT	1.279 (1.111–1.427)	0.001	NS	NS
INR	10.285 (2.609–40.554)	0.001	NS	NS
aPTT	1.102 (1.059–1.146)	<0.001	1.074 (1.021–1.130)	0.005
Fibrinogen^a^	1.959 (1.668–2.300)	<0.001		
D-dimer	1.065 (1.022–1.011)	<0.001	NS	NS
High FAR (FAR≥0.0977)	4.341 (2.775–6.791)	<0.001	2.830 (1.654–4.840)	<0.001
Anticoagulant drugs	1.782 (1.057–3.003)	0.03	NS	NS
Antiplatelet drugs	0.241 (0.126–0.461)	<0.001	NS	NS
Length of stay	1.137 (1.083–1.193)	<0.001	NS	NS

*Logistic regression analysis adjusted for the 12 variables (P < 0.1) listed. SAP, stroke-associated pneumonia; OR, odds radio; FAR, fibrinogen to albumin ratio; PT, prothrombin time; aPTT, activated partial thromboplastin time; INR, international normalized ratio*.

a *These parameters are not entered in the multivariate analysis in order to prevent multicollinearity*.

**Figure 4:**
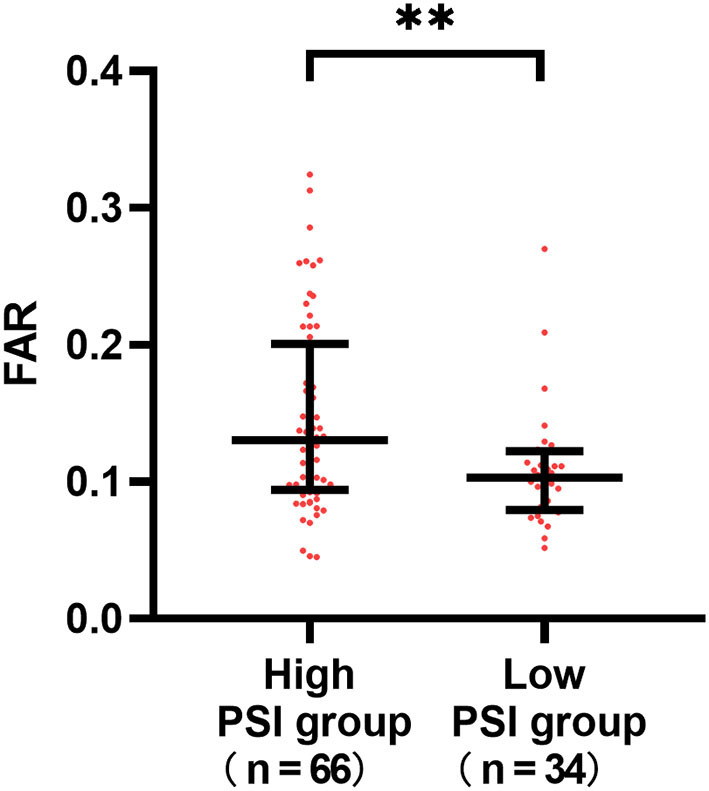
Differences in the fibrinogen to albumin (FAR) according to the severity of stroke-associated pneumonia (SAP). The high PSI group (0.13 [0.94–0.19] vs. 0.10 [0.79–0.12]; *P* = 0.008) had significantly higher FARs than the low scoring groups. **P < 0.01.

To assess the relationship between pneumonia severity and the FAR, we analyzed 100 patients with SAP (median FAR: 0.113[0.909–0.167]). The high PSI group (0.13 [0.94–0.19] vs. 0.10 [0.79–0.12]; *P* = 0.008) had significantly higher FARs than the low scoring groups, showing a close correlation between the FAR and pneumonia severity (Figure 4).

## Discussion

To the best of our knowledge, this is the first study to explore the role of FAR in the development of SAP. In this retrospective study, we found that coagulation functions differ significantly between patients with SAP and non-SAP. Patients with SAP had a higher level of fibrinogen, PT, aPTT, D-dimer, and lower albumin levels at admission. We also found an independent relationship between higher FAR in the first hours after hospitalization and the development of SAP after adjusting for potential contributory factors. This suggested the possible utility of FAR as a marker for the risk of developing SAP. In addition, high FAR levels correlated with pneumonia severity. Furthermore, our data demonstrated that FAR could effectively predict the incidence of SAP with an optimal cutoff point of 0.0977 that could identify the risk of SAP, with remarkable sensitivity and specificity. Therefore, FAR could be a useful and valid biomarker to identify potential patients with SAP at admission.

Compared with other literature, our study first described the coagulation parameters between SAP and non-SAP and found high FAR was an independent risk for the prevalence of SAP. In our study, 100 (10.7%) patients were diagnosed with SAP, which was consistent with previous reports (6). The study showed age, baseline NIHSS score, AF, dysphagia, and length of hospital stay were contributory factors toward SAP, which was also consistent with previous reports (9). In addition, our study showed that antiplatelet therapy was related to a low risk of SAP. The link has not been studied yet. Studies regarding coagulation functions and SAP are rare. In an animal experiment carried out on models of acute lung injury, researchers found nebulized heparin could reduce pulmonary coagulopathy and inflammation effectively by modulating alveolar macrophages (31). During the COVID-19 pandemic, Bi et al. found that fibrinogen, FAR, and D-dimer were significantly elevated in severe patients with COVID-19 with inferior prognosis and accelerated clinical progression (32). A recent study found high levels of D-dimer could increase the risk of 3-month mortality and death/severe disability in AF-related patients with AIS (33). More evidence indicates that an extensive link exists between coagulation and inflammation. The acute phase of inflammation response leads to excessive activation of the coagulation system, and coagulation also amplifies the inflammation process considerably (34, 35). Despite this, the effect of FAR on acute patients with IS, of whom a high proportion suffer from coagulation dysfunction, had not been studied until now.

As fibrinogen and albumin, two critical factors in the coagulation system that care for biomarkers for nutrition state and inflammation, are delivered by the liver, the combined application of two factors (FAR) could be a more powerful predictor than single biomarker fibrinogen or albumin. An elevated FAR level may be a result of increased fibrinogen levels or/and decreased albumin levels. In this study, the FAR levels of patients with SAP were higher than patients with non-SAP (0.113 vs. 0.087, *P* < 0.001; Table 1). The FAR level of patients with hemorrhagic transformation (HT) was higher than the patients with non-HT after stroke in a previous study (8.60 vs. 10.29, respectively; Table 1) (36). This may be due to the inflammatory response caused by the occurrence of stroke. When the immune system participates in the ischemic brain processes, stroke-induced immunodepression can protect our body from excessive inflammatory reactions. However, it promotes the risk of concurrent infections and affects the prognosis of stroke patients (37). Nonetheless, the association between FAR and AIS requires further study.

Fibrinogen, known as a 340 kDa glycoprotein, can be deposited within and around damaged tissue zones. In research conducted in Spain, Miranda Acuña et al. found elevated fibrinogen deposition in the brain in a wide range of neurological diseases such as multiple sclerosis (38). However, the underlying mechanisms between fibrinogen and SAP remain unclear. There could be a potential mechanism to explain this association. First, fibrinogen is identified as a ligand for various cell surface receptors. It could serve as an intermediate molecule to boost cell-to-cell adhesion between leukocytes and the endothelium. In this way, fibrinogen can facilitate the migration of leukocytes outside the blood vessel to specific tissues (39, 40), and induce changes in leukocytes function. This can take place through cell movement, phagocytosis, release of chemokines and cytokines, degranulation, and nuclear factor-Kappa B-mediated transcription (41–45). Second, plasma fibrinogen levels increase by 2–3 times in response to inflammation, causing cell aggregation, higher blood viscosity, and an increase in endothelin-1 (46). Moreover, during the fibrinolysis process, plasmin, the major fibrinolytic protease, can degrade extracellular matrix proteins and activates matrix metalloproteinases resulting in tissue damage (47, 48).

Serum albumin levels represent the state of nutrition status of an individual and indicate the presence of inflammation. Inflammation increases capillary permeability and flee of serum albumin, causing the distribution volume of albumin to increase while total albumin mass decreases. Albumin accumulates at inflammation sites so it has been widely used as an ideal drug delivery platform in anti-inflammation therapy (49). Several studies revealed that low serum albumin was considered a high-risk factor for deterioration and poor prognosis in patients with nosocomial infection (50, 51). Growing research evidence showed that albumin was a useful, independent prognostic factor in patients after stroke (52, 53). In a cohort study of 759 patients with AIS followed for 3 months, a poor outcome was independently related to low serum albumin level, ischemic heart disease, and infarction size (53). Based on physiological characteristics of albumin, namely, antioxidant, anti-inflammatory, anticoagulant, and antiplatelet aggregation activity and regulation of microvascular permeability, albumin could be used to predict the pulmonary infection after stroke.

Notably, FAR baseline levels correlated with poststroke pneumonia during hospitalization. It also seems plausible that there may be an association between FAR and pneumonia severity, although inflammation response changes dynamically overtime during AIS. It is interesting that this relationship continued despite the effects of the inflammation process.

There remained some limitations in our study. First, our study was based on a single-center retrospective database and some inevitable subjective selections bias might have existed, reducing the reliability of the investigation. Second, FAR was only recorded once at admission and could not be recorded at other times (beyond 24 h), which limited the analysis of the dynamic association between FAR and SAP. It is necessary to conduct a further longitudinal study to verify the predictive value of FAR measured at multiple times for SAP. Third, our study lacked data on the use of the nasogastric tubes and mechanical ventilation, which were related to a high risk of respiratory infections. Finally, other important blood parameters such as antithrombin and leukocyte were not included in this study.

## Conclusions

Despite the limitations, our findings are very meaningful. Our study revealed that high FAR was an independent predictive factor related to a higher risk of SAP in patients with AIS. It was also related to the severity of pneumonia. The study provides new references for further clinical trials to determine whether the levels of FAR may serve as a new marker to select patients with AIS at a higher risk of SAP and thus, to guide targeted treatment at an early stage.

## Data Availability Statement

The original contributions presented in the study are included in the article/supplementary material, further inquiries can be directed to the corresponding author/s.

## Ethics Statement

The studies involving human participants were reviewed and approved by the Ethics Committee of First Affiliated Hospital of Wenzhou Medical University. Written informed consent for participation was not required for this study in accordance with the national legislation and the institutional requirements.

## Author Contributions

GL, MH, JS, ZW, and JH conceived and designed the study. GL and MH interpreted data. GL, MH, and JS wrote the manuscript. GL, JS, and XX prepared figures. GL and JS did the statistical analyses. GL, MH, JS, XX, HL, LQ, HZ, MX, DG, and LY screened and extracted data. ZW, JH, and GH supervised the study. All authors have made an intellectual contribution to the manuscript and approved the submission.

## Funding

This work was supported by the Projects of Provincial Natural Science Foundation of Zhejiang (no. LY19H090013), the Jiaxing Science and Technology Plan Project (no. 2019AD32181) and Institute of Aging, Key Laboratory of Alzheimer's Disease of Zhejiang Province, Wenzhou Medical University, Wenzhou, Zhejiang, China.

## Conflict of Interest

The authors declare that the research was conducted in the absence of any commercial or financial relationships that could be construed as a potential conflict of interest.

## Publisher's Note

All claims expressed in this article are solely those of the authors and do not necessarily represent those of their affiliated organizations, or those of the publisher, the editors and the reviewers. Any product that may be evaluated in this article, or claim that may be made by its manufacturer, is not guaranteed or endorsed by the publisher.

## Abbreviations

AIS: acute ischemic stroke
PSM: propensity score matching
AUROC: area under the receiver operating characteristic curve
OR: odds ratio
FAR: fibrinogen to albumin ratio
SBP: systolic blood pressure
DBP: diastolic blood pressure
NIHSS: National Institute of Health Stroke Scale
AF: atrial fibrillation
mRS: modified Rankin Scale.
